# The first seven years of nationally organized helicopter emergency medical services in Finland – the data from quality registry

**DOI:** 10.1186/s13049-020-00739-4

**Published:** 2020-05-29

**Authors:** Anssi Saviluoto, Johannes Björkman, Anna Olkinuora, Ilkka Virkkunen, Hetti Kirves, Piritta Setälä, Ilkka Pulkkinen, Päivi Laukkanen-Nevala, Lasse Raatiniemi, Helena Jäntti, Timo Iirola, Jouni Nurmi

**Affiliations:** 1 Research and Development Unit, FinnHEMS, WTC Helsinki Airport, Lentäjäntie 3, FI-01530 Vantaa, Finland; 2 University of Eastern Finland, PO Box 1627, FI-70211 Kuopio, Finland; 3 University of Helsinki, PO Box 4, FI-00014 Helsinki, Finland; 4 Prehospital Emergency Care, Hyvinkää hospital area, Hospital District of Helsinki and Uusimaa, PO Box 585, FI-05850 Hyvinkää, Finland; 5 Emergency Medical Services, Tampere University Hospital, PO Box 2000, FI-33521 Tampere, Finland; 6 Centre for Prehospital Emergency Care, Oulu University Hospital, PO Box 50, FI-90029 Oulu, Finland; 7 Center for Prehospital Emergency Care, Kuopio University Hospital, PO Box 100, FI-70029 Kuopio, Finland; 8 Emergency Medical Services, Turku University Hospital and University of Turku, PO Box 52, FI-20521 Turku, Finland; 9 Emergency Medicine and Services, Helsinki University Hospital and Emergency Medicine, University of Helsinki, PO Box 100, FI-00029 Helsinki, Finland

**Keywords:** Air ambulances, Emergency medical services, Critical care, Registries, Trends, Quality indicators

## Abstract

**Background:**

Helicopter Emergency Medical Services (HEMS) play an important role in prehospital care of the critically ill. Differences in funding, crew composition, dispatch criteria and mission profile make comparison between systems challenging. Several systems incorporate databases for quality control, performance evaluation and scientific purposes. FinnHEMS database was incorporated for such purposes following the national organization of HEMS in Finland 2012. The aims of this study are to describe information recorded in the database, data collection, and operational characteristics of Finnish HEMS during 2012–2018.

**Methods:**

All dispatches of the six Finnish HEMS units recorded in the national database from 2012 to 2018 were included in this observational registry study. Five of the units are physician staffed, and all are on call 24/7. The database follows a template for uniform reporting in physician staffed pre-hospital services, exceeding the recommended variables of relevant guidelines.

**Results:**

The study included 100,482 dispatches, resulting in 33,844 (34%) patient contacts. Variables were recorded with little or no missing data. A total of 16,045 patients (16%) were escorted by HEMS to hospital, of which 2239 (2%) by helicopter. Of encountered patients 4195 (4%) were declared deceased on scene. The number of denied or cancelled dispatches was 66,638 (66%). The majority of patients were male (21,185, 63%), and the median age was 57.7 years. The median American Society of Anesthesiologists Physical Scale classification was 2 and Eastern Cooperative Oncology Group performance class 0. The most common reason for response was trauma representing 26% (8897) of the patients, followed by out-of-hospital cardiac arrest 20% (6900), acute neurological reason excluding stroke 13% (4366) and intoxication and related psychiatric conditions 10% (3318). Blunt trauma (86%, 7653) predominated in the trauma classification.

**Conclusions:**

Gathering detailed and comprehensive data nationally on all HEMS missions is feasible. A national database provides valuable insights into where the operation of HEMS could be improved. We observed a high number of cancelled or denied missions and a low percentage of patients transported by helicopter. The medical problem of encountered patients also differs from comparable systems.

## Background

Helicopter Emergency Medical Services (HEMS) play an important role in prehospital care of the critically ill in many Emergency Medical Services (EMS) around the world. However, little is known about when and where the utilization of this expensive resource is beneficial [1–3]. Organization of HEMS varies considerably between countries and states. Differences in funding, composition of crew, emergency dispatch, patient population, hospital network and geographical characteristics make the applicability of study results and comparison between services challenging [3]. Consequently, accurate and comprehensive data is a prerequisite for the development and improvement of any service [4].

Quality registries are a valuable source of data when evaluating the performance of any field in health care [4, 5]. For this purpose, several HEMS systems utilize databases to collect and analyze mission data [6–8]. To enable multi-center research and comparison between systems, guidelines for data collection have been published for prehospital airway management [9, 10]., cardiac arrest [11, 12]. and physician-staffed emergency medical services (P-EMS) [13, 14]. Since 2012 HEMS operations have been nationally organized. At the start of the national service, a database called FinnHEMS database (FHDB) was established to record detailed information on all HEMS missions in the country. FHDB has been adjusted to conform with the previously stated guidelines, but it also stores additional information not required by the guidelines.

Owing to a nationally organized and documented HEMS, FHDB gives a comprehensive view of the whole system. The aims of this study are 1) to describe the data collected into FHDB and evaluate the completeness of the data. 2) To describe the operational and patient characteristics of HEMS in Finland during the first 7 years of nationally organized and documented HEMS.

## Methods

### Study design

This was an observational registry study, describing all HEMS dispatches in Finland during 1.1.2012–31.12.2018**.** Study permission was requested and granted by all the participant hospital districts (Oulu University Hospital 200/2019 2.7.2019, Helsinki University Hospital HUS/280/2019 9.7.2019, Turku University Hospital J30/19 4.8.2019, Hospital District of Lapland 32/2019 22.8.2019, Kuopio University Hospital RPL 102/2019 22.8.2019, Tampere University Hospital RTL-R19580 2.9.2019). According to Finnish Law, ethical permission is not required for observational studies. However, due to the large amount of data, including sensitive patient data, ethical permission was requested and granted by the Ethical Board of the University of Helsinki (HUS/3115/2019 §194). STROBE guidelines are followed in reporting of the study [15].

### Setting

Finland is a Nordic country with a population of 5.5 million and an area of 338,424 km^2^, thus making it the most sparsely populated country in the European Union. Healthcare is governed regionally by 21 hospital districts, each braced by one of the five university hospitals for tertiary care.

EMS in Finland are locally organized by the hospital districts. The system is publicly funded, including the dispatch centers and HEMS. Since 2012 HEMS is administered by a national administrative unit FinnHEMS Ltd., owned and governed by the five university hospitals during the study period. A few central hospitals have also organized physician staffed rapid response cars to support the local EMS.

Emergency calls are all made to a national emergency number 112 (healthcare, fire, police & social services). The calls are processed by Emergency Response Centre Operators (ERCO) in one of the six regional dispatch centers using a nationally unified, tiered dispatch structure assisting in the dispatch of units, with slight local variations. ERCOs are specially educated for the task but they are not healthcare providers. In addition to being alarmed by the dispatch centers, the HEMS units can also be requested by the EMS crews. A list of dispatch codes and those leading to HEMS activation can be seen in Additional File 1.

HEMS units are alerted to patients who are thought to benefit from early prehospital intensive care. Typical alarm criteria are Out-of-Hospital Cardiac Arrest (OHCA), major trauma and unconsciousness with an unknown origin. In the Finnish EMS system, HEMS units are not normally dispatched to conscious stroke patients, patients suffering from respiratory failure, and cardiovascular accidents, with the exception of the unit based in Lapland, due to the extremely sparse population and long distances in the area.

**Fig. 1:**
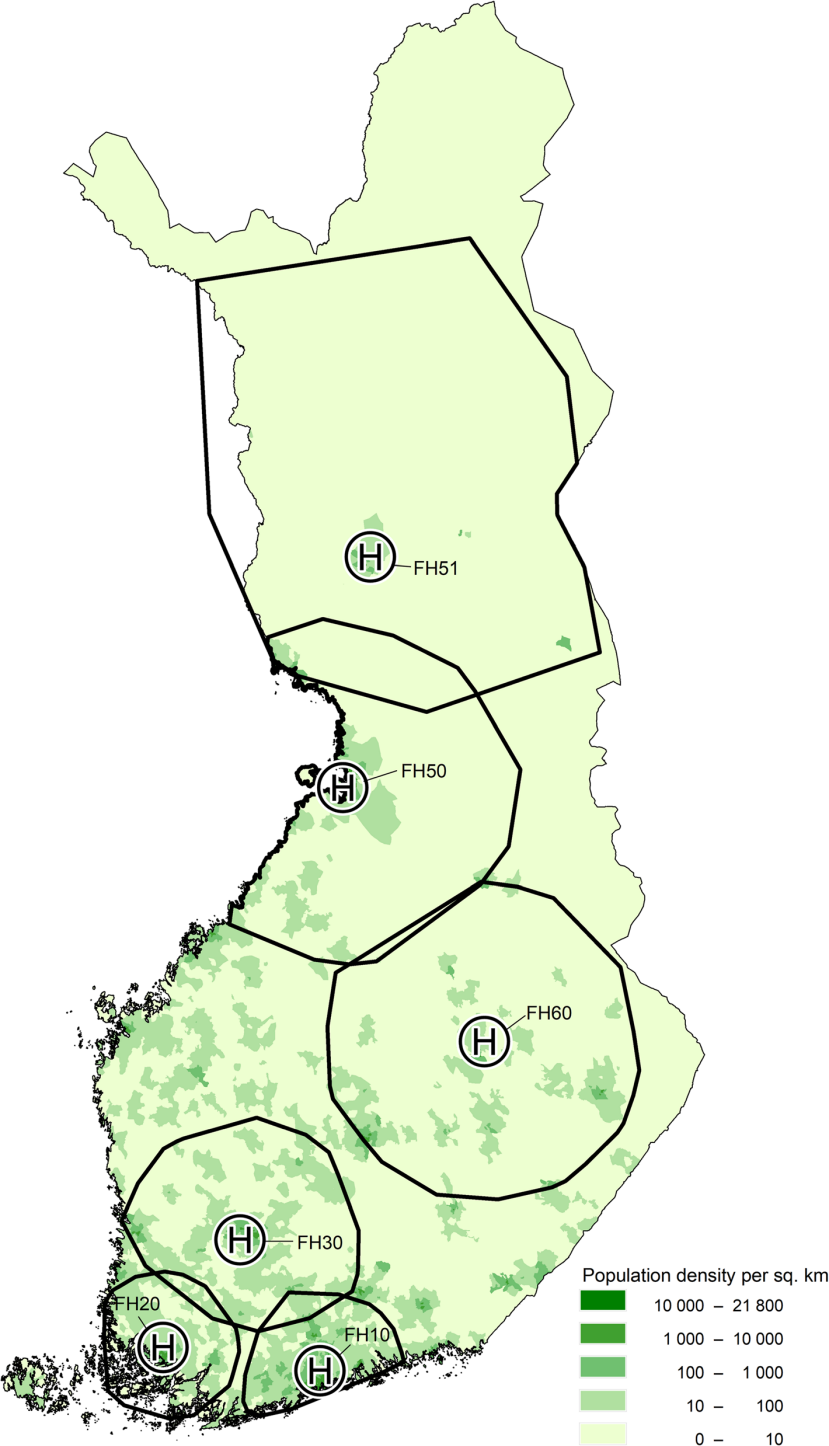
The population density of Finland, location of HEMS bases and their actual service areas with 95% of the missions in 2017 [16, 17]. The population density is shown as density (population per km^2^) per postal area. H = HEMS base, FH = FinnHEMS unit

Five HEMS units are based at the university hospitals and one in Lapland, and their actual service areas encompassing 95% of the operations cover nearly the whole population of Finland (Fig. 1) [16, 17]. The three southernmost units operate with Airbus H135 and the three other units with Airbus H145 helicopters. The primary task of the HEMS units is prehospital care, with rare interhospital transfers and search-and-rescues being decided upon in a case-by-case fashion.

The HEMS units operating out of the university hospitals are staffed by a physician, a HEMS Crew Member (HCM; either a paramedic or a firefighter according to local regulations) and a pilot, while the unit in Lapland has two advanced level flight paramedics and two pilots. Physicians in the HEMS units are mainly anesthesiologists with sub specialization in prehospital critical care whereas HCMs are specially trained in prehospital critical care as well as in aviation. The HEMS pilots have significant previous experience in either civilian or military helicopter operations.

The wide array of equipment and medications used in the HEMS units is not nationally standardized but locally governed by the hospital districts. All units are on-call 24/7/365 and are capable of flying under instrument flight rules and night-time flight operations using night vision goggles. Rapid response vehicles are available for the HEMS crews in every base for short-range missions or for when weather conditions don’t meet the HEMS minima for airborne operations.

### FinnHEMS database

As stated previously, the data variables recorded in FHDB follow a template for uniform reporting in physician staffed pre-hospital services according to relevant guidelines [9–14]. A list of the current central variables and their response rate is shown in Additional File 2, also depicting compulsory variables. Over the years there have been minor revisions in the datasets, presented in Additional File 3. FHDB is used for daily reporting, scientific purposes and the governing of HEMS operations as a whole [16, 18–20]. The database also allows the creation of specific case-report forms for research projects or to monitor effects of specific quality improvement interventions. The database does not allow extremely abnormal values that are clearly erroneous, but it does not interfere with single erroneous input that are within the normal variation range. Therefore, all input to the database was seen as valid, and clearly erroneous input was also included, since this study describes the database per se and validity thereof, not the actual operational set. In addition, some variables are in selected cases recorded by the first ambulance unit at the scene, which also might affect the validity. During the initial years of FHDB key entered data was not mandatory, this has since been rectified. The data is entered by the physician or paramedic on call promptly following a mission, using a web-based form over a secure connection. However, the entered data is not externally validated by any other person, making errors a possibility. Data input in the web-form is immediately recorded to FHDB.

### Statistical methods

Normally and non-normally distributed continuous data are reported as means with standard deviations (SD) and medians with quartiles (25th percentile and 75th percentile expressed as Q1/Q3), respectively. Categorical variables are reported as percentages with 95% confidence intervals (95% CI). Proportions are reported as % (n). As this was a descriptive analysis of the entries recorded to the database, no comparisons between any groups were necessary. The data was analyzed using IBM SPSS Statistics 25 (IBM Corporation, Armonk, NY, USA).

## Results

### Subjects

**Fig. 2:**
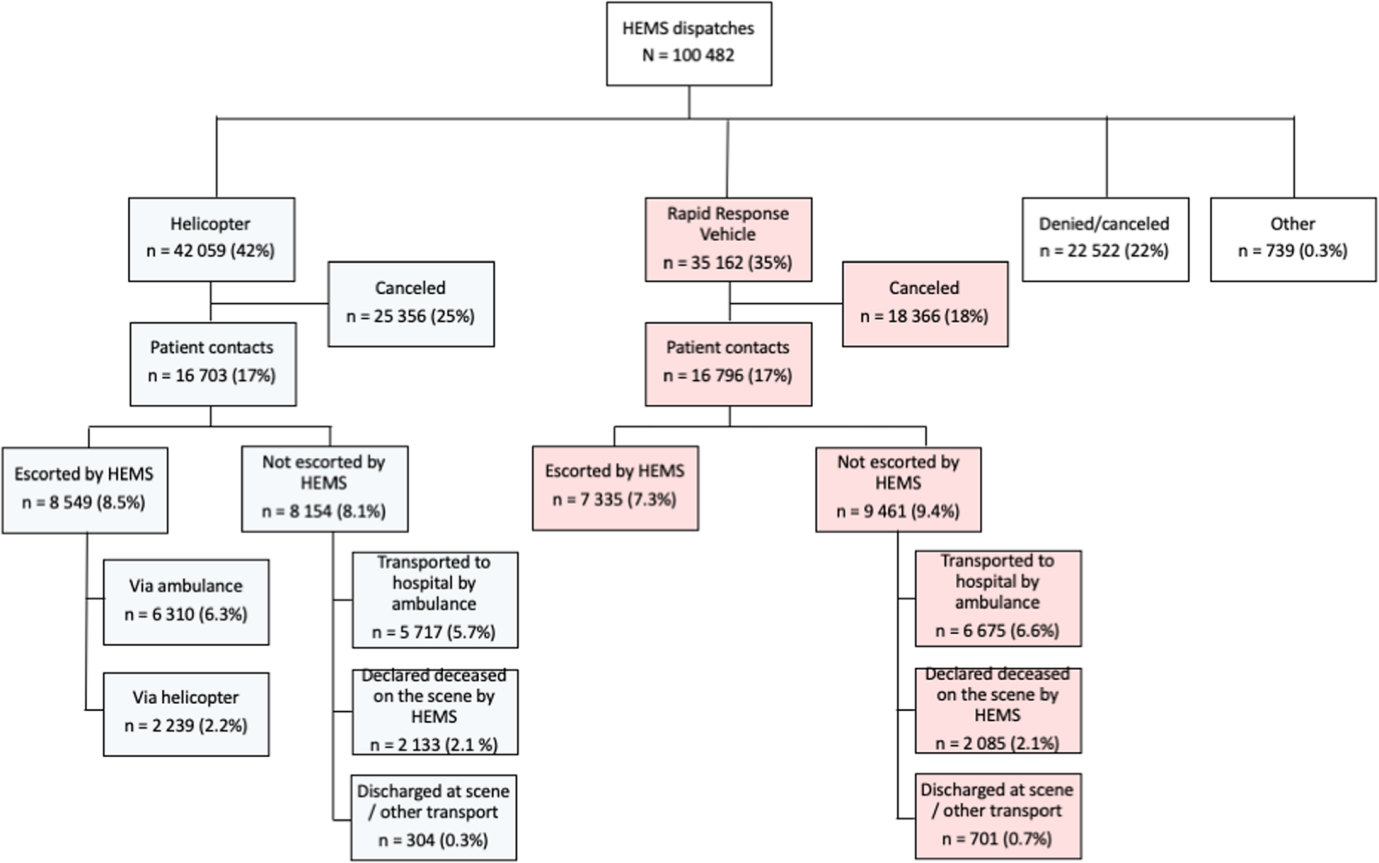
Diagram of HEMS dispatches. Revisions to FHDB makes it unreliable to discern denied missions from cancelled directly following an alarm. Missions canceled after start of the mission are labelled as “canceled”. Percentages are of total N

All 100,482 dispatches recorded into the FHDB between 1.1.2012–31.12.2018 were included in this study (Fig. 2). Multi-patient dispatches (*n* = 569, 0.6%) were analyzed as one entry per dispatch and not per patient.

### Operational mission characteristics

Of the accepted dispatches, 54% (42,059) were responded to with a helicopter and 45% (35,162) with a rapid response vehicle. In the remaining 1% (739) another mode of transport was used (e.g. Border Guard Helicopter, other vehicle). The main reasons for rapid response vehicle use was short distance (51%, 18,103) or weather below HEMS minima criteria (35%, 12,437).

**Fig. 3:**
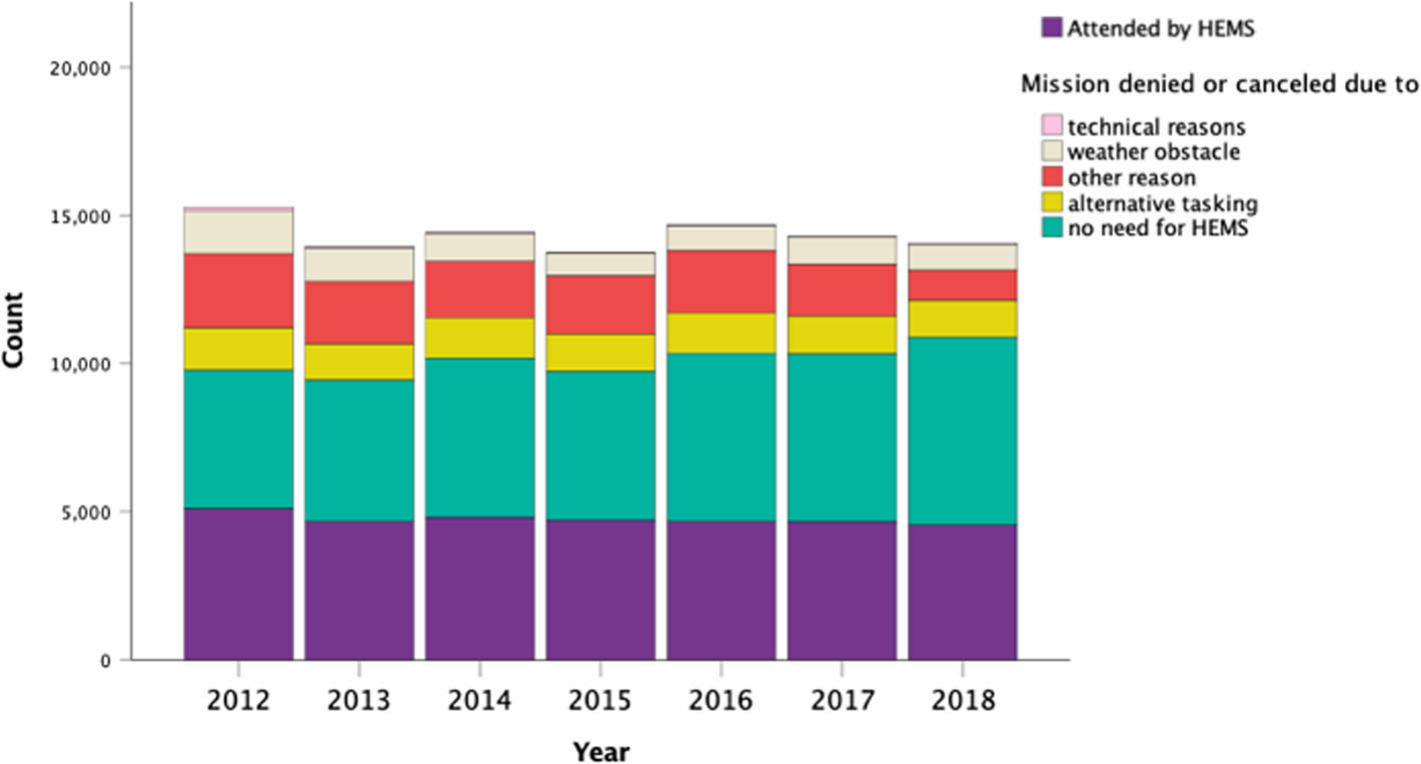
Annual change in the outcome of the HEMS dispatches

A total of 67% (66,638) of the dispatches were denied or cancelled. These included dispatches that were denied or cancelled due to not requiring HEMS care after additional information on status of the patient was received (56%, 37,542), denied or cancelled due to alternative tasking (14%, 9163), denied or cancelled due to weather (10%, 6950) or denied or cancelled due to technical obstacle (0.5%, 344), the remaining 20% being recorded as “denied or cancelled due to other reason”. Annual changes in the proportions are shown in Fig. 3.

**Fig. 4:**
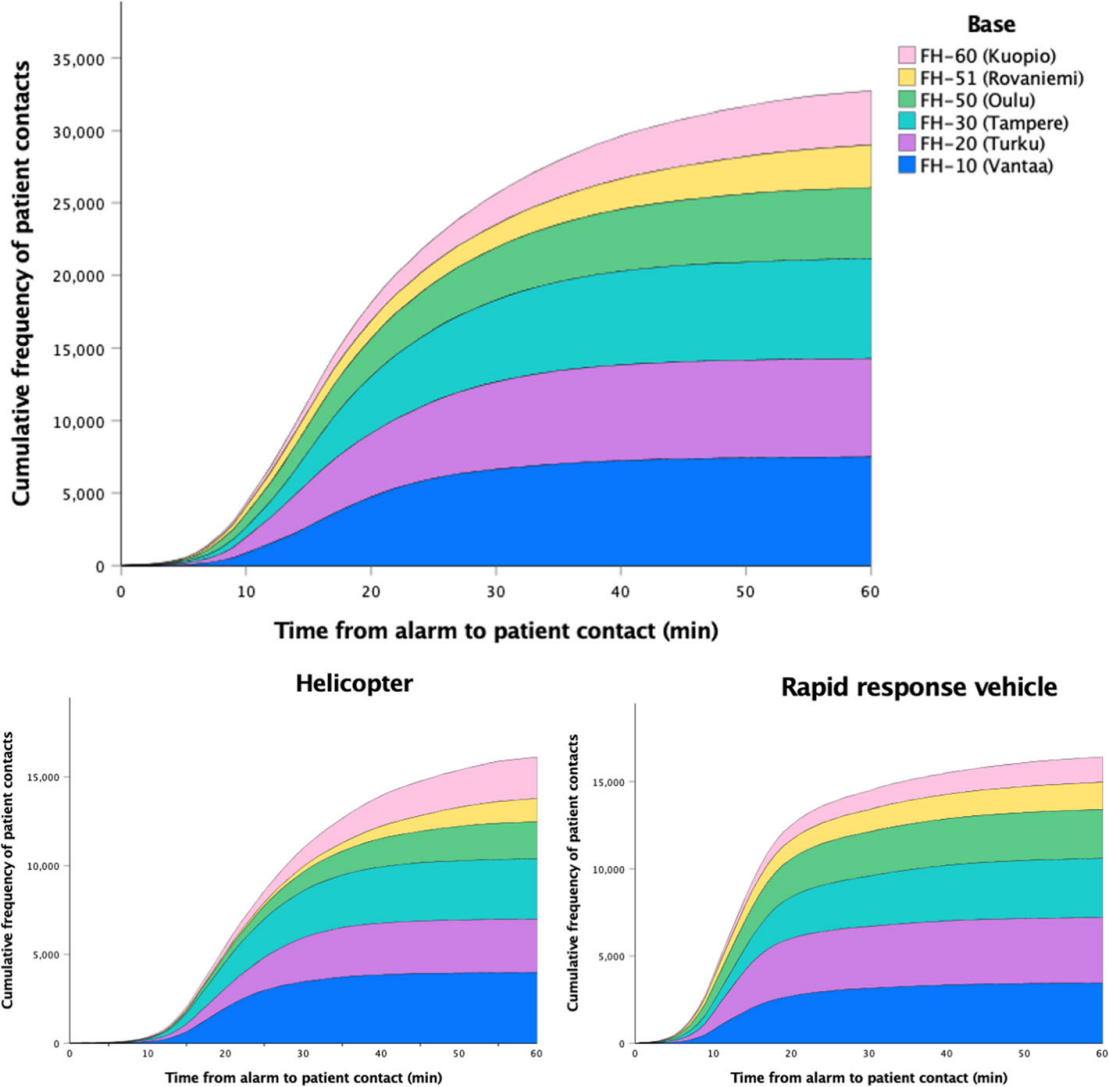
The cumulative frequency distribution of response time in HEMS bases during 2012–2018 (*n* = 33,844). The median was 25 min when responding with the helicopter and 15 min with the rapid response vehicle

Response time from alarm to patient contact was available for all missions where the patient was encountered, and the median was 19 min (Q1/Q3 14/30 min, Fig. 4). On-scene time was available in 94.7% (15,255) of the missions where the patient was either escorted or transported by HEMS, the median being 23 min (Q1/Q3 12/36 min). Correspondingly, the transport time was available for 92.8% (14,969) of all dispatches with a median of 25 min (Q1/Q3 14/41 min).

### Patient characteristics

**Fig. 5:**
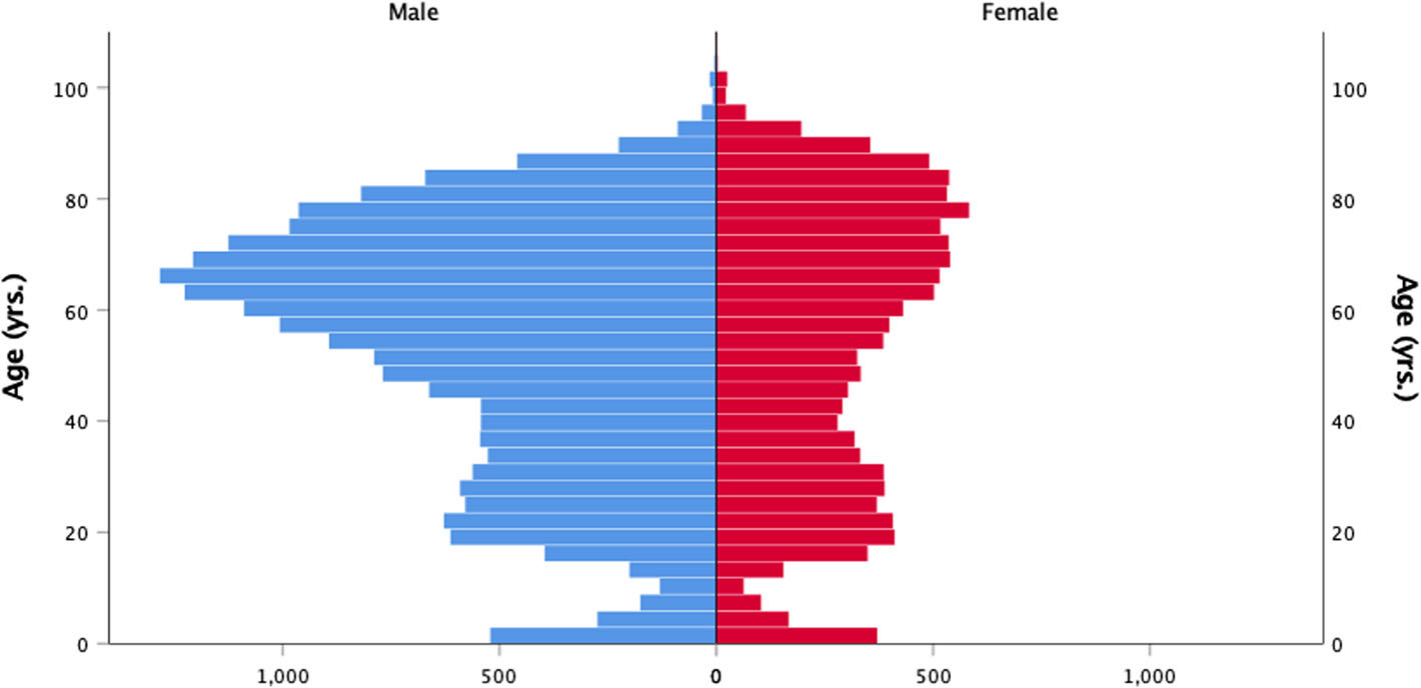
Age and gender of patients encountered by HEMS

Of the patients encountered by HEMS, 35% (12,011) were females and 63% (21,185) males, with gender reported as “not known” in 2% (648) of missions. The median age of the patients was 57.7 years (Q1/Q3 33.8/72.0 years) (Fig. 5).

American Society of Anesthesiologists Physical Scale (ASA-PS) classification was available for 91% (30,477) and Eastern Cooperative Oncology Group (ECOG) performance classification - made mandatory in the database in 2014 - was available for 90% (21,397) of the patients, the rest being recorded as “unknown”. Most patients were classified in ASA-PS classes I and II (64%) and ECOG classes 0 and 1 (82%) (Table 1).

**Table 1:** Patient characteristics (*n* = 33,820). ASA-PS Class = American Society of Anesthesiologist Physical Status, ECOG = Eastern Cooperative Oncology Group. Q1/Q3 denotes 25th and 75th percentiles

	n	(%)	Median	Q1/Q3	Missing n	(%)
Age, years	33,820		57.7	33.8/72.2	24	(0.07)
Gender, female	12,011	(35.5)			648	(1.9)
ASA-PS Class	30,477		2	1/3	3367	(9.9)
I	10,422	(30.8)				
II	9083	(26.8)				
III	8098	(23.9				
IV	2491	(7.4)				
V	303	(0.9)				
VI	80	(0.2)				
ECOG	21,397		0	0/1	2367	(10.0)
0	13,489	(18.9)				
1	4126	(5.8)				
2	2114	(3.0)				
3	1375	(1.9)				
4	293	(0.4)				

**Fig. 6:**
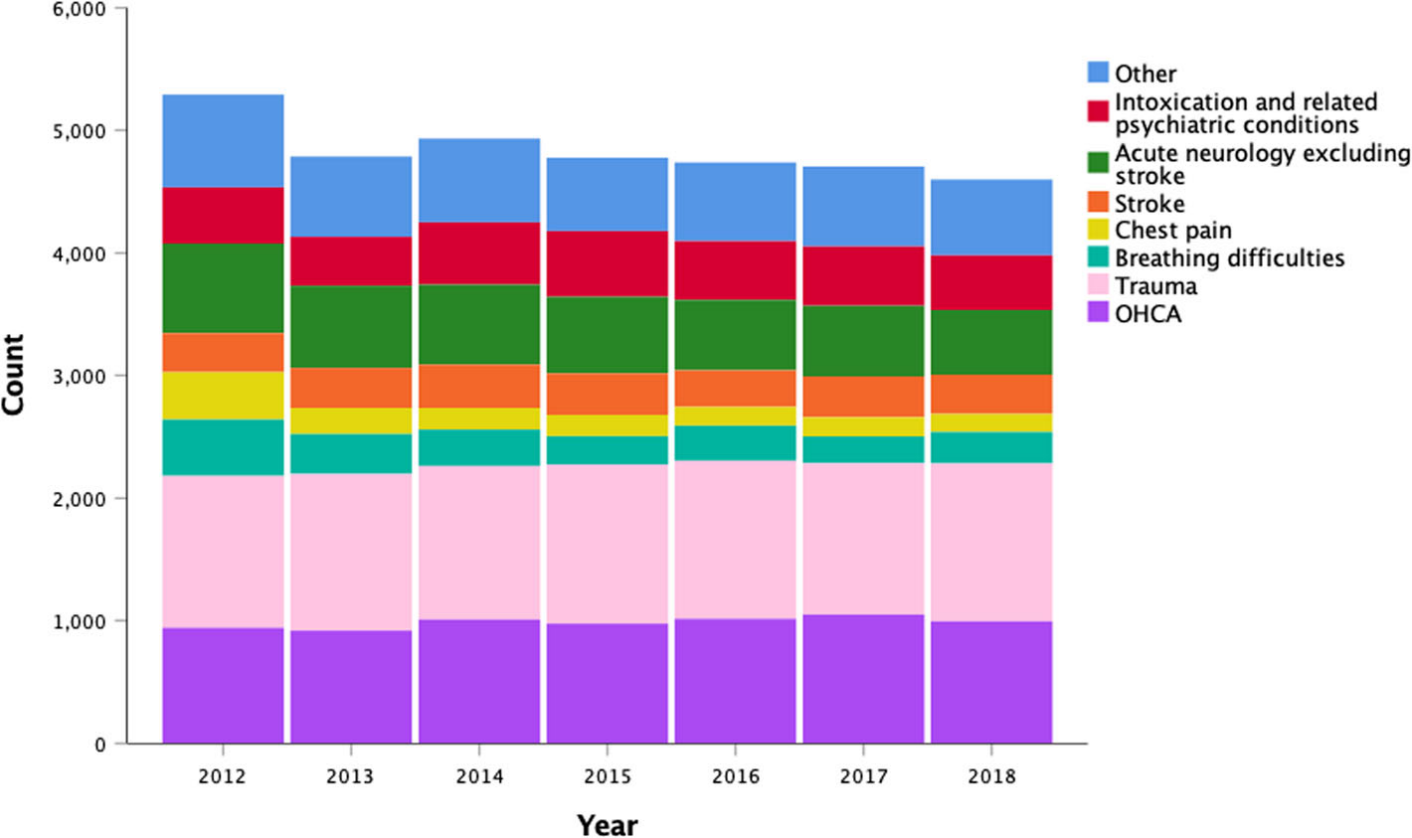
The medical problem reported for patients encountered by HEMS. OHCA denotes out-of-hospital cardiac arrest

The medical problem was reported in all missions leading to patient contact with temporal changes represented in Fig. 6. The most common reason for response was trauma in 26% (8897) of the missions, followed by OHCA in 20% (6900) and acute neurological reason excluding stroke, and intoxication and related psychiatric conditions in 13% (4366) and 10% (3318) respectively. Of the trauma, 86% (7653) were classified as blunt and 13% (1141) as penetrating. For the remaining 1% (103) data was recorded as “Not Classified” or “Other”.

## Discussion

In this study we established that gathering detailed and comprehensive data nationally on all HEMS missions while closely adhering to relevant guidelines is feasible with low rates of missing data. Key findings of this study were that in the Finnish HEMS system a large proportion of missions are cancelled or denied, and a relatively small percentage of patients are transported by helicopter. We also found that the medical problem for HEMS dispatch differ from comparable systems [7, 8, 21].

In this study we concluded that it was not possible to evaluate the validity of the recorded data. Data is entered manually by the paramedic or physician on the mission and thus errors are acceptable. This should be kept in mind when using this data. Dealing with extreme values and possible recording errors have to be taken into account in study designs. Where recording errors are suspected the data can be compared to the original documentation of the EMS or HEMS unit on-scene. This method could also be used for a future study to assess the validity of the data. This does not exclude hidden errors such as heart rate input as 96/min when the correct value is 69/min. In the future electronic transfer of measurements could be used to reduce typing errors, however, this does not eliminate the errors in measurement per se.

The rate of cancelled or denied missions in the Finnish HEMS system is substantially higher than reported elsewhere [6, 7, 21, 22]. Technical reliability of the service seems to be excellent and it contributed to the cancellation rate minimally. Indeed, most often the cancellation was based on the HEMS physician’s judgement, following the information available from ERCO and EMS unit on the mission, that interventions by the HEMS team were not needed or that the patient would not benefit from these due to severe comorbidities etc. For patients requiring fast access to critical care to be adequately recognized, a certain amount of overtriage is unavoidable, but excessive overtriage may lead to increased costs and missing simultaneous patients that could have benefitted from HEMS [23]. Having the HEMS physician decide on a large amount of cancellations may also lead to decision fatigue [24, 25]., increasing the risk for inappropriate cancellations [25]. The effectiveness of the service could potentially be increased by improving dispatch criteria or by flight paramedic interrogation of the caller [26, 27].

Secondly, transporting a patient by helicopter in Finland is rare compared to other services [6–8]. Use of helicopter can provide significant time saving [28, 29]. with the added benefit of being able to bypass a local hospital and flying directly to an appropriate tertiary-care center [30]. HEMS has been used to transport trauma patients for a long time, but advances in invasive endovascular therapies have increased the use of aeromedical transport for patients with stroke or myocardial infarction as well. When distances are short, a rapid response vehicle is used in Finland instead of a helicopter, partly explaining the difference compared to the systems operating only by helicopter to the longer distances. Three of the six bases in Finland are located in mostly urban areas where distances are generally short and the use of helicopter might not result in time saving [31, 32]. As shown by our study, the practice of escorting a patient transported in an ambulance is more common in our system compared to other systems [6–8]. However, several studies have demonstrated benefit from timesaving by helicopter transportation in select patient groups [28, 29, 31]., raising the question whether helicopter transportation should be more frequent.

Our study also revealed marked disparities in medical problem for dispatch compared to other countries. Similarly to other systems, trauma formed the largest subset of patients. Trauma is indeed the most common cause of preventable death in the previously healthy [33]., and several studies have found HEMS to provide benefit to this specific patient group [34–36]. The second most common medical problem was OHCA, representing a large proportion compared to other systems [7, 8, 21]. A study in the Finnish population supports dispatching HEMS for OHCA [37]., but it remains largely unclear whether it incurs a survival benefit in this group [2]. Contrary to other comparable systems where stroke and myocardial infarction are common reasons for HEMS dispatch [7, 8, 21]., Finnish physician-staffed HEMS units are not usually dispatched for these missions, although encounter these patients when dispatched on different criteria, such as decreased level of consciousness. There is evidence suggesting that stroke and MI patients might stand to benefit from primary dispatch of HEMS [29, 31].

To provide actionable information, the data in a quality registry must be comprehensive [4]. Overall, missions recorded in FHDB had low levels of missing data. Vital signs (listed in Additional file 2) were made compulsory at the end of 2013, after which missing data rate has been very low. The initial years account for almost all of the missing data in vital signs. During this time period, missing values might be more common in critically ill patients introducing bias. However, the database offers information that can be used to assess this sort of bias, such as the physician’s assessment on the seriousness of the patient’s conditions. If need be, data between 2012 and 2013 could be excluded altogether. No other significant lack of values in clinical parameters were observed.

To enable multi-center research projects and comparison between systems the data has to conform to international standards. Therefore, several revisions have been made to FHDB to include variables recommended by guidelines (see Additional File 3). Most of the guidelines, e.g. airway template [9]., are followed precisely. However, not all of the core variables in the Utstein reporting template for cardiac arrest [11]. are included in FHDB as they are more appropriate to a regional cardiac arrest registry than a HEMS quality register. Despite these guidelines and increased interest in HEMS, the literature on HEMS databases is scarce and comparison between systems is challenging.

### Strengths and limitations

A nationally administrated HEMS enables the maintenance of a shared, uniform database, constituting a strength of this study. The data is collected prospectively and recorded recently after mission conclusion, and the database includes every single Finnish HEMS dispatch since 2012. FinnHEMS Ltd. is funded by the government and the HEMS units are dispatched solely on medical criteria without insurance policy, wealth or socioeconomic status of the patient biasing patient selection.

As previously mentioned, the data is manually entered and not independently validated and is therefore prone to subjective factors or errors. During the existence of the database there have been necessary revisions on how certain variables are recorded or classified, making the analysis of trends before and after these changes challenging. Some of the recorded variables are highly subjective resulting in varying levels of disparities between providers as in any system collecting such variables, but overall a previous study found acceptable rates of inter-rater variability [20].

Patient selection limits the generalizability of the results. The database contains only a subset of all EMS patients and dispatching criteria in our system might omit patient groups prominent in other systems. Despite these limitations, FHDB stores a large amount of nationwide data that can be, and already has been, used for further research in prehospital EMS and HEMS [16, 18, 19]. The data is being combined with the national discharge register and mortality data, enabling studies assessing survival, factors associated with changes in mortality and length of hospitalization. Several such studies are already in progress.

## Conclusions

Gathering detailed and comprehensive data nationally on all HEMS missions and treated patients is feasible. A national database provides valuable insights into where the operation of HEMS could be improved. We observed a high number of cancelled or denied missions, highlighting the need for more accurate dispatching. The low use of helicopter transportation compared to other services suggests that there might be a need to re-evaluate the current practice. Nonetheless, the medical problem of encountered patients also differs from comparable systems.

## Supplementary information

**Additional file 1.** Dispatch codes leading to HEMS-activation

**Additional file 2.** Central variables in FHDB

**Additional file 3.** Revisions to the FHDB

## Data Availability

An anonymized dataset with relevant variables supporting the findings may be requested for from the corresponding author.

## Abbreviations

HEMS: Helicopter emergency medical services
EMS: Emergency medical services
P-EMS: Physician-staffed emergency medical services
FHDB: FinnHEMS database
ERCO: Emergency response centre operator
OHCA: Out-of-hospital cardiac arrest
FH: FinnHEMS unit
HCM: HEMS crew member
SD: Standard deviation
Q: Quartile
CI: Confidence interval
ASA-PS: American Society of Anesthesiologists Physical Scale
ECOG: Eastern Cooperative Oncology Group
