# Supplementary material for: The first seven years of nationally organized helicopter emergency medical services in Finland – the data from quality registry
Source: Scand J Trauma Resusc Emerg Med. 2020 May 29;28:46. doi: 10.1186/s13049-020-00739-4 (PMC7260827; doi:10.1186/s13049-020-00739-4)
Supplement: Supplementary file 1 — Additional file 1. Dispatch codes leading to HEMS-activation [file 13049_2020_739_MOESM1_ESM.docx]

## Additional File 1

# Dispatch codes leading to HEMS-activation

Emergency response centre operators assign a code for each mission with an urgency rating (A/B/C/D) with A being the most urgent. HEMS-units are alerted for the depicted codes when the urgency is rated A. Due to long distances, the HEMS unit in Lapland (FH51) is alerted for additional codes, including some when the urgency rating is B. Other minor differences exist between HEMS-units due to differences in geography and local health care systems. In accidents, HEMS is dispatched only in case of casualties.

| **Code** | **All HEMS-units** | **Lapland HEMS only** |
| --- | --- | --- |
| 700 sudden cardiac arrest | X |  |
| 701 resuscitation (request for back-up in cardiac arrests confirmed by EMS or rescue) | X |  |
| 702 loss of consciousness | X |  |
| 703 breathing difficulty, dyspnea |  | X |
| 704 chest pain |  | X |
| 705 arrythmia |  | X |
| 706 stroke |  | X (B) |
| 707 urgent interhospital transfer |  | X (B) * |
| 711 airway blockage/obstruction | X |  |
| 713 hanging, strangulation | X |  |
| 714 submersion | X |  |
| 741 fall from height | X |  |
| 744 wound | X |  |
| 745 fall on same level | X |  |
| 746 mechanical impact | X |  |
| 747 other mechanical trauma | X |  |
| 751 gas exposure | X |  |
| 752 intoxication | X |  |
| 753 electric shock | X |  |
| 755 burns, hyperthermia | X |  |
| 756 hypothermia | X |  |
| 757 other trauma caused by exposure |  |  |
| 761 hemorrhage, oral |  | X |
| 762 hemorrhage, gynecological/urological |  | X |
| 763 hemorrhage, ear/nose |  |  |
| 764 leg ulcer/other hemorrhage |  |  |
| 770 unspecified medical emergency |  |  |
| 771 diabetic disorders |  |  |
| 772 convulsions |  | X |
| 773 anaphylaxis |  | X |
| 774 weakened general condition, other illness |  |  |
| 775 vomiting, diarrhea |  |  |
| 781 abdominal pain |  | X |
| 782 head/neck pain |  | X |
| 783 back/hip pain |  |  |
| 784 limb pain |  |  |
| 785 psychiatric disorder |  |  |
| 786 pain in area of torso |  |  |
| 790 non-specified urgent dispatch during emergency call |  |  |
| 791 childbirth | X |  |
| 012 mass casualty incident | X |  |
| 031 shooting | X |  |
| 032 stabbing | X |  |
| 033 assault | X |  |
| 200-204 traffic accident (different types) | X |  |
| 210-217 railway accident (different types) | X |  |
| 222-223 waterway accident (different types) | X |  |
| 231-233 air traffic accident (different types) | X |  |
| 234-236 air traffic hazard (different types) | X |  |
| 271 motor vehicle accident, off-road (snowmobile, ATV etc.) | X | X (B) |
| 401-403 burning building (different types) | X |  |
| 441-443 explosion/caving (different types) | X |  |
| 451-453 chemical accident (different types) | X |  |
| 483 rescue from water | X |  |
| 486 rescue from being crushed | X |  |

*Not automatically assigned but decided on a case-by-case basis.

(B) Unit is assigned when the urgency is rated B in addition to a rating of A.
