# Supplementary material for: The first seven years of nationally organized helicopter emergency medical services in Finland – the data from quality registry
Source: Scand J Trauma Resusc Emerg Med. 2020 May 29;28:46. doi: 10.1186/s13049-020-00739-4 (PMC7260827; doi:10.1186/s13049-020-00739-4)
Supplement: Supplementary file 2 — Additional file 2. Central variables in FHDB [file 13049_2020_739_MOESM2_ESM.docx]

## Additional file 2

# Central variables in FHDB

|  | **Response rate %** | **n** | **Missing n** |
| --- | --- | --- | --- |
| **Dispatch information** |  |  |  |
| Dispatch ID | 100,0 | 100482 |  |
| Patient ID | 100,0 | 33844 |  |
| Base | 100,0 | 100482 | 0 |
| Date of dispatch | 100,0 | 100482 | 0 |
| Time of dispatch | 100,0 | 100482 | 0 |
| Dispatch code | 100,0 | 100482 | 0 |
| City of residence | 100,0 | 33844 | 0 |
| Care facility | 100,0 | 100482 | 0 |
| Care facility type | 100,0 | 8357 | 0 |
| Treatment limitation | 100,0 | 100482 | 0 |
| Type of treatment limitation |  | 923 |  |
| City of dispatch | 100,0 | 100482 | 0 |
| Hospital district of dispatch | 100,0 | 100482 | 0 |
| Dispatcher | 100,0 | 100482 | 0 |
|  |  |  |  |
| **Unit information** |  |  |  |
| Crew composition | 100,0 | 100482 | 0 |
| Response vehicle type | 100,0 | 100482 | 0 |
| Reason for ground unit use | 100,0 | 100482 | 0 |
| Effect on prognosis of ground unit use | 100,0 | 100482 | 0 |
| Transportation code for HEMS unit | 100,0 | 100482 | 0 |
| Reason for cancellation | 99,5 | 66276 | 362 |
| Reason for delay |  | 4863 |  |
| Ambulance unit | 33,7 | 33839 | 66643 |
| Location at alarm | 99,9 | 100373 | 109 |
|  |  |  |  |
| **Timestamps** |  |  |  |
| Time from alarm to mobile | 88,7 | 69133 | 8827 |
| Time of emergency call | 62,6 | 62944 | 37538 |
| Time of Dispatch | 100,0 | 100482 | 0 |
| Time mobile | 88,7 | 69134 | 8826 |
| Time cancelled | 66,0 | 43754 | 22522 |
| Time at scene | 100,0 | 34156 |  |
| Time at patient | 98,4 | 33289 | 555 |
| Time transport started | 95,7 | 15410 | 695 |
| Time at hospital | 93,7 | 15084 | 1021 |
| Time available for new mission | 100,0 | 34844 | 0 |
| Time at mission end | 100,0 | 100482 | 0 |
| Time from dispatch to patient | 100,0 | 33844 | 0 |
| On-scene time | 94,7 | 15255 | 850 |
| Transport time | 92,9 | 14964 | 1141 |
| Total time | 100,0 | 100482 | 0 |
| Patient encountered during transport |  | 6530 |  |
|  |  |  |  |
| **First responder information** |  |  |  |
| Type of first responder | 100,0 | 33844 | 0 |
| First responder dispatch time | 100,0 | 1051 |  |
| First responder at scene time | 100,0 | 938 |  |
| Ambulance dispatch time | 100,0 | 29927 |  |
| Ambulance at scene time | 100,0 | 19838 |  |
|  |  |  |  |
| **Patient and transport** |  |  |  |
| No of patients | 100,0 | 100482 | 0 |
| HEMS Benefit Score | 100,0 | 33844 | 0 |
| Gender | 100,0 | 100482 | 0 |
| Age | 99,9 | 33820 | 24 |
| ICD10 diagnosis^*^ | 44,2 | 14974 | 18870 |
| ICPC-2 code^*^ | 55,8 | 18870 | 14974 |
| Transporting unit | 100,0 | 33844 | 0 |
| Transportation code | 100,0 | 33844 | 0 |
| Escorted by HEMS | 100,0 | 33844 | 0 |
| Receiving hospital | 100,0 | 16101 | 4 |
| Survival to hospital | 73,7 | 8523 | 3039 |
| Logistic advantage of helicopter transport |  | 1053 |  |
| Mission outcome | 100,0 | 100482 | 0 |
|  |  |  |  |
| **Interventions and physiological variables** |  |  |  |
| Co-morbidity | 90,1 | 30477 | 3367 |
| Performance status | 90,0 | 21397 | 2367 |
| Medical problem | 100,0 | 33844 | 0 |
| Dominating type of injury | 100,0 | 8953 |  |
| Drugs to facilitate airway procedure |  |  |  |
| - Anaesthetic | 100,0 | 33844 | 0 |
| - Neuromuscular blocking agent | 100,0 | 33844 | 0 |
| - Analgesic/opioid | 100,0 | 33844 | 0 |
| - Local/topical anaesthetic | 100,0 | 33844 | 0 |
| Breathing procedures used |  |  |  |
| - Assisted manually | 100,0 | 33844 | 0 |
| - Assisted mechanically | 100,0 | 33844 | 0 |
| - Controlled manually | 100,0 | 33844 | 0 |
| - Controlled mechanically | 100,0 | 33844 | 0 |
| - Chest tube/decompression | 100,0 | 33844 | 0 |
| - Thoracostomy | 100,0 | 33844 | 0 |
| Circulation procedures used |  |  |  |
| - Peripheral IV line | 100,0 | 33844 | 0 |
| - Central IV line | 100,0 | 33844 | 0 |
| - IO access | 100,0 | 33844 | 0 |
| - Defibrillation | 100,0 | 33844 | 0 |
| - Cardioversion | 100,0 | 33844 | 0 |
| - External pacing | 100,0 | 33844 | 0 |
| - Haemostatic, basic | 100,0 | 33844 | 0 |
| - Haemostatic, advanced | 100,0 | 33844 | 0 |
| - Other circulatory procedures | 100,0 | 33844 | 0 |
| Disability procedures |  |  |  |
| - Reduction of fractures | 100,0 | 33844 | 0 |
| - Spinal immobilisation | 100,0 | 33844 | 0 |
| - Therapeutic hypothermia | 100,0 | 33844 | 0 |
| - Other disability procedures | 100,0 | 33844 | 0 |
| Medication |  |  |  |
| - Analgesic/opioid | 100,0 | 33844 | 0 |
| - Anaesthetic/sedative | 100,0 | 33844 | 0 |
| - Neuromuscular blocking agent | 100,0 | 33844 | 0 |
| - Vasoactive | 100,0 | 33844 | 0 |
| - Fibrinolytic | 100,0 | 33844 | 0 |
| - Antibiotic | 100,0 | 33844 | 0 |
| - Other medication | 100,0 | 33844 | 0 |
| Diagnostic procedures |  |  |  |
| - US/Doppler | 100,0 | 33844 | 0 |
| - ECG-analysis (12-lead) | 100,0 | 33844 | 0 |
| - Invasive monitoring | 100,0 | 33844 | 0 |
| - Point-of-care laboratory tests | 100,0 | 33844 | 0 |
| - Other diagnostic procedures | 100,0 | 33844 | 0 |
| Cardiac rhythm-first | 91,8 | 31081 | 2763 |
| Heart rate-first | 90,0 | 30473 | 3371 |
| Systolic BP-First | 90,0 | 30451 | 3393 |
| Respiratory rate-first | 77,0 | 26064 | 7780 |
| SpO2-first | 89,5 | 30304 | 3540 |
| EtCO2-first | 72,6 | 24554 | 9290 |
| Cardiac rhythm-last | 85,5 | 15171 | 2568 |
| Heart rate-last | 86,7 | 15375 | 2364 |
| Systolic BP-last | 87,3 | 15486 | 2253 |
| Respiratory rate-last | 72,5 | 12866 | 4873 |
| SpO2-last | 87,5 | 15521 | 2218 |
| EtCO2-last | 70,5 | 12509 | 5230 |
| GCS eyes-first | 92,9 | 31430 | 2414 |
| GCS verbal response-first | 92,9 | 31425 | 2419 |
| GCS motor response-first | 92,9 | 31426 | 2418 |
| GCS total-first | 93,9 | 31768 | 2076 |
|  |  |  |  |
| **Resuscitation** |  |  |  |
| Time of OHCA |  | 6383 |  |
| Temporary ROSC |  | 1515 |  |
| Final ROSC |  | 3139 |  |
|  |  |  |  |
| **Airway** |  |  |  |
| Airway management | 100,0 | 33844 | 0 |
| Airway indication | 100,0 | 7324 |  |
| Airway secured by | 100,0 | 7324 | 0 |
| Airway intervention attempts | 98,1 | 7188 | 136 |
| Airway intervention success | 98,1 | 7188 | 136 |
| Airway complications |  |  |  |
| - Oesophageal intubation | 98,2 | 7191 | 133 |
| - Main stem intubation | 98,2 | 7191 | 133 |
| - Dental damage | 98,2 | 7191 | 133 |
| - Vomit/regurgitation | 98,2 | 7191 | 133 |
| - Hypoxia | 98,2 | 7191 | 133 |
| - Bradycardia | 98,2 | 7191 | 133 |
| - Hypotension | 98,2 | 7191 | 133 |
| Failed intubation |  | 987 |  |
|  |  |  |  |

* Diagnose coding system changed during from ICD-10 to ICPC2
