# Supplementary material for: The first seven years of nationally organized helicopter emergency medical services in Finland – the data from quality registry
Source: Scand J Trauma Resusc Emerg Med. 2020 May 29;28:46. doi: 10.1186/s13049-020-00739-4 (PMC7260827; doi:10.1186/s13049-020-00739-4)
Supplement: Supplementary file 3 — Additional file 3. Revisions to the FHDB [file 13049_2020_739_MOESM3_ESM.docx]

Additional file 3

# Revisions to the FHDB

Central changes and additions to the variables recorded to the database.

| 12/2012 | Added | Respiratory rate-first and Respiratory rate-last |
| --- | --- | --- |
| 12/2012 | Added | EtCO2-first and EtCO2-last |
| 02/2013 | Added | Time from alarm to mobile and Reason for delay |
| 06/2013 | Added | Patient encountered during transport |
| 12/2013 | Added | Care facility type |
| 12/2013 | Added | Performance status |
| 12/2013 | Added | Airway indication, airway indication (text), airway secured by, Airway intervention attempts, Airway intervention success and Airway complication-esophageal intubation |
| 12/2013 | Added | GCS, respiratory, heart rate, blood pressure, saturation and EtCO2 before and after intervention |
| 12/2013 | Added | Respiratory rate-after intervention and Respiratory rate-at hospital |
| 10/2014 | Removed | Primary ICD10 diagnosis and Secondary ICD10 diagnosis |
| 10/2014 | Added | Primary ICPC-2 code, Secondary ICPC-2 code and 3D-locator for trauma |
| 12/2014 | Added | Failed intubation |
| 03/2016 | Removed | Survival to hospital moved from airway sheet |
| 03/2016 | Added | Survival to hospital |
| 03/2016 | Added | Logistic advantage of helicopter transport |
| 03/2016 | Added | Debriefing template |
| 06/2016 | Added | NQM study |
| 06/2016 | Removed | NQM study |
| 12/2016 | Added | Freezing flying conditions |
| 05/2017 | Added | Cognitive load and strain |
| 08/2017 | Added | Treatment limitation types revised |
| 08/2017 | Added | Category of treatment limitation |
| 01/2018 | Removed | Freezing flying conditions |
| 04/2018 | Removed | Treatment limitation types revised |
| 06/2018 | Removed | Cognitive load and strain |
| 10/2018 | Removed | Airway intervention attempts, failed intubation and Airway intervention success |
| 10/2018 | Added | Revision of Airway template |
| 10/2018 | Added | Time of death |
| 11/2018 | Added | Circulatory template revised |
| 11/2018 | Added | Mechanical resuscitation assist device used |
| 11/2018 | Added | Temperature variables revised |
